# Enhancement of postprandial endogenous insulin secretion rather than exogenous insulin injection ameliorated insulin antibody-induced unstable diabetes: a case report

**DOI:** 10.1186/s12902-018-0326-3

**Published:** 2019-01-08

**Authors:** Keizo Kaneko, Chihiro Satake, Tomohito Izumi, Mamiko Tanaka, Junpei Yamamoto, Yoichiro Asai, Shojiro Sawada, Junta Imai, Tetsuya Yamada, Hideki Katagiri

**Affiliations:** Department of Diabetes and Metabolism, Tohoku University Hospital, 2-1 Seiryo, Aoba-ku, Sendai, Miyagi 980-8575 Japan

**Keywords:** Hypoglycemia, Insulin antibody, Insulin autoimmune syndrome, Postprandial endogenous insulin

## Abstract

**Background:**

Insulin injection, especially with insulin analogs, occasionally induces the production of insulin antibodies with high binding capacity and low affinity, similar to the insulin autoantibodies characteristic of insulin autoimmune syndrome (IAS). Production of these “IAS-like” insulin antibodies causes marked glycemic fluctuations with postprandial hyperglycemia and fasting hypoglycemia.

**Case presentation:**

A 66-year-old man with a 27-year history of diabetes was admitted because of marked glycemic fluctuations. Human insulin treatment had been initiated at age 56, followed by multiple daily injections of insulin analogs 5 years later. After the initial year of insulin analog treatment, the patient began to experience frequent morning hypoglycemic attacks and day-time hyperglycemia. Marked hyperinsulinemia (4500 μU/mL) and high titers of insulin antibodies (80.4%) with high binding capacity and low affinity indicated that IAS-like insulin antibodies were causing severe glucose fluctuations. Altering insulin formulations (insulin aspart → regular human insulin→ insulin lispro) proved to be ineffective. After several therapeutic trials, cessation of exogenous insulin and addition of mitiglinide to liraglutide with voglibose finally attenuated glycemic fluctuations with increased postprandial insulin secretion. Continuous glucose monitoring revealed improvement of morning hypoglycemia and postprandial hyperglycemia with smaller mean amplitude of glycemic excursion. Therefore, compared to exogenously injected insulin, endogenously secreted insulin directly and rapidly acts on hepatocytes and suppresses postprandial glucose output.

**Conclusions:**

Proper enhancement of postprandial endogenous insulin aimed at suppressing postprandial glucose output without stimulating excessive glucose uptake in the periphery is potentially useful for treating diabetes with insulin antibody-induced glycemic instability.

## Background

Insulin autoimmune syndrome (IAS), or Hirata’s disease, is characterized by the presence of insulin-binding autoantibodies in patients who have never been exposed to exogenous insulin [1]. Scatchard plot analysis has revealed that this type of insulin autoantibody has high binding capacity and low affinity [1], thereby causing remarkable hyperinsulinemia and fasting or late postprandial hypoglycemia. Eating small meals and α-glucosidase treatment to decrease insulin secretion are recommended to suppress the hyperinsulinemia [2]. Exogenously injected insulin can also trigger the production of insulin antibodies, but these antibodies mostly do not disturb glycemic controls [3]. However, it has recently been reported that insulin antibodies occasionally result in characteristics similar to those due to insulin autoantibodies in IAS patients, including marked glycemic fluctuations with postprandial hyperglycemia and fasting hypoglycemia [4–6].

In these patients, changing insulin formulations is occasionally effective for decreasing insulin antibody levels and improving glycemic control [7]. However, we experienced an insulin-treated patient with the “IAS-like” insulin antibodies whose glycemic fluctuations were not reduced by altering insulin formulations. We speculated that subcutaneous administration of exogenous insulin has two potential disadvantages compared to endogenous insulin secreted from pancreatic β cells. First, exogenously injected insulin persisting at subcutaneous sites may have more chances to be captured by insulin antibodies. Second, endogenously secreted insulin directly and rapidly acts on hepatocytes and suppresses postprandial glucose output from the liver, while subcutaneously injected insulin may exacerbate hypoglycemia due to exerting preferential actions on muscle and fat tissues. Based on these ideas, we switched the treatment from insulin injections to mitiglinide with liraglutide and voglibose with the aim of enhancing postprandial secretion of endogenous insulin, and finally attenuated the glycemic fluctuations.

## Case presentation

A 66-year-old man with a 27-year history of diabetes was hospitalized because of severe morning hypoglycemia and postprandial hyperglycemia.

At age 39, he had shown symptoms of polyuria and was diagnosed as having type 2 diabetes. At the initial treatment, an allergic skin reaction to porcine insulin occurred, requiring a switch from insulin to oral hypoglycemic agents. He often missed clinic visits, which contributed to very poor glycemic control. He was then hospitalized for glycemic control (HbA1c: 11.7% (104 mmol/mol)) when he was 54 years old. During this hospitalization, his fasting serum C-peptide level was 1.41 ng/mL and blood glucose levels were promptly improved by dietary treatment with oral hypoglycemic agents (sulfonylurea and α-glucosidase). He underwent photocoagulation therapy for proliferative-diabetic retinopathy and his creatinine level was 1.0 mg/dL at this time.

At age 56, premixed human insulin 30/70 was administered after an episode of diabetic ketoacidosis with subcutaneous abscesses, but, again, due mainly to his poor adherence, his glycemic control had remained very poor with HbA1c of approximately 10.0% (86 mmol/mol). Then, a complete cessation of treatment for three years resulted in a marked HbA1c increase to 18.9% (183 mmol/mol). At this point (61 years old), multiple daily insulin therapy using insulin analogs, i.e. aspart before each meal and detemir before bedtime, was introduced and his HbA1c levels gradually decreased. However, after one year of treatment with insulin analogs, hypoglycemic attacks in the morning manifested. In addition, postprandial hyperglycemia developed and his severe glycemic fluctuations were not reduced by switching basal insulin from detemir to degludec and glargine. The plasma creatinine level was maintained at approximately 1.0 mg/dL with proteinuria for three years after the beginning of the hypoglycemic episodes, but had recently risen to nearly 2.0 mg/dL. He had hypertension and developed peripheral artery disease with mild claudication. To prevent exacerbation of vascular complications, the patient has been treated with an angiotensin II receptor blocker, since age 64 years. He was diagnosed as having hypothyroidism with negative thyroid antibodies and had been treated with levothyroxine since age 65 years. To examine the mechanism underlying his glycemic fluctuations and to devise an effective treatment strategy, he was admitted to our hospital.

**Fig. 1:**
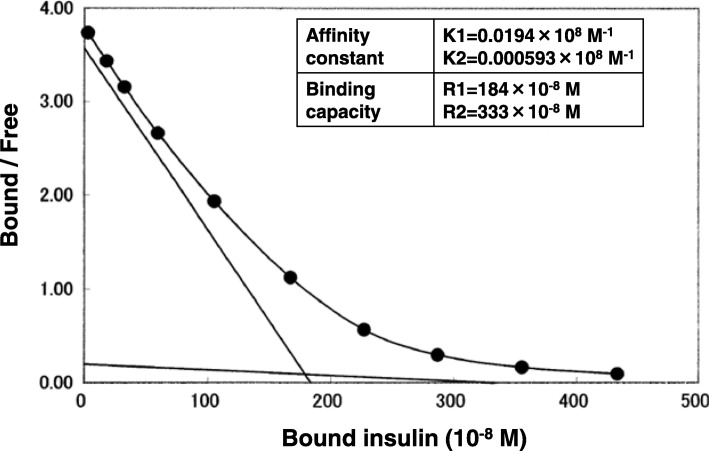
The Scatchard plot analysis of the ^125^I-insulin binding study was performed by SRL, Inc., Tokyo, Japan. K1 and R1 values of the antibody represent, respectively, the affinity constant and binding capacity of the high affinity sites of the antibody. K2 and R2 values of the antibody represent, respectively, the affinity constant and binding capacity of the low affinity sites of the antibody

On admission, his fasting blood glucose level and HbA1c were 62 mg/dL (3.4 mmol/L) and 9.3% (82 mmol/mol), respectively. Total insulin level was extremely elevated at 4500 μU/mL. The total insulin values represented both endogenous and exogenous insulin because not only human insulin but also recombinant insulin analogs cross-react with the ARCHITECT® insulin assay (Abbott Laboratories) [8]. He was positive for insulin antibodies with the binding rate being 80.4% and Scatchard plot analysis (on the 2nd day of hospitalization) revealed 0.0194 × 10^8^ M^− 1^ (K1) and 184 × 10^− 8^ M (R1) (Fig. 1), indicating high binding capacity and low affinity. The characteristics were similar to those of the autoantibodies reported in IAS patients. His HLA (human leukocyte antigen) haplotypes were HLA-DRB1* 040101/ HLA-DRB1*0406 and HLA-DQB1*030201/ HLA-DQB1*050101 and, among them, HLA-DRB1*0406 is associated with the highest risk for susceptibility to IAS [9]. Insulin-specific IgE antibody and islet-related autoantibodies, such as those against glutamic acid decarboxylase (GAD) and insulinoma-associated antigen-2 (IA-2) were undetectable. Counter regulatory hormones against insulin action were not lowered (Table 1). Estimated glomerular filtration rates were between 13 and 25 mL/min/1.73m^2^, showing chronic renal failure. Serum hepatic enzyme levels were within normal ranges. Neither computed tomography nor magnetic resonance imaging revealed any tumors in the pancreas, excluding the existence of insulinoma. Taking these findings together, the IAS-like insulin antibodies were considered to be the cause of glycemic instability in this patient.

**Table 1:** Laboratory Data

		Unit	Normal rage
HbA1c	9.1 (76)	% (mmol/mol)	4.6–6.2
Insulin antibody	80.4	%	0–0.4
Insulin-specific IgE	< 0.01	UA/mL	0
GAD andtibody	< 0.3	U/mL	0–1.4
IA-2 antibody	< 0.4	U/mL	0–0.4
Glucagon	144	pg/mL	71–174
Adrenaline	< 0.05	ng/mL	0–0.1
Noradrenaline	0.41	ng/mL	0.1–4.5
Cortisol	18.9	μg/dL	6.2–19.4
ACTH	30.5	pg/mL	4.4–48
GH	2.79	ng/mL	0–2.47
Insulin-like growth factor-Ι	73	ng/mL	70–229
Aspartate aminotransferase	35	IU/L	8–38
Alanine aminotransferase	21	IU/L	4–43
γ-glutamyl transferase	28	IU/L	10–47
Blood urea nitrogen	29	mg/dL	8–20
Creatinine	2.16	mg/dL	0.44–1.15
HLA typing	DRB1*04:01:01/ DRB1*04:06 DQB1*03:02:01/ DQB1*05:01:01		

*GAD* glutamic acid decarboxylase, *IA-2* insulinoma-associated antigen-2, *ACTH* adrenocorticotropic hormone, *GH* growth hormone, *HLA* human leukocyte antigen

First, to prevent the frequent hypoglycemic attacks, we repeatedly adjusted the treatment regimen (Table 2). We stopped insulin glargine before bedtime, followed by cessation of insulin aspart before each meal. At 36 h after the cessation of all insulin administrations, insulin levels fell to 2500 μU/mL and glucose levels were elevated and remained over 360 mg/dL (20 mmol/L) throughout the day, indicating supplemental insulin, either exogenous or endogenous, to be necessary for prevention of hyperglycemia. Next, bolus insulin, restarted with altered formulations comprised of regular human insulin or insulin lispro, inhibited neither postprandial hyperglycemia nor morning hypoglycemia. Therefore, instead of increasing exogenous insulin, we administered other agents to selectively suppress postprandial hyperglycemia.

**Table 2:** Fasting glucose and insulin levels with corresponding treatment regimens

Day after hospitalization	FG mg/dL (mmol/L)	Insulin μU/mL	Treatment regimen (Analysis by CGM) [Analysis of C-peptide responses to each meal]
1			Asp 4–4-4, stopped insulin glargine
2	62 (3.4)	4500	
3	47 (2.6)		
5	55 (3.1)		
8			Stopped Asp from dinner
10	358 (19.9)	2500	
16	362 (20.1)		Start Asp 4–6-4
20	61 (3.4)		Stopped Asp, start RHI 4–4-4
30			RHI 4–4-4, add LIRA 0.3 mg QD
38	77 (4.3)		RHI 4–4-4 + LIRA 0.6 mg QD
41			RHI 3–3-3 + LIRA 0.9 mg QD
43	65 (3.6)	3570	
45	79 (4.4)		Stopped RHI, start Lis 3–3-3 + LIRA 0.9 mg QD
51	52 (2.9)		Lis 3–3-3 + LIRA 0.9 mg QD, add Voglibose 0.2 mg QD
53	51 (2.8)	3500	(CGM in Fig. 2a)
54			[C-peptide measurement in Fig. 3]
56	63 (3.5)		Lis 2–3-3 + LIRA 0.9 mg QD + Voglibose 0.2 mg QD
58	68 (3.8)		Lis 2–3-3 + LIRA 0.9 mg QD + Voglibose 0.2 mg QD, add Mitiglinide 10 mg TID
59	46 (2.6)		LIRA 0.9 mg QD + Voglibose 0.2 mg QD + Mitiglinide 10 mg TID, stopped Lis [C-peptide measurement in Fig. 3]
61	91 (5.1)		
63	95 (5.3)		(CGM in Fig.2b)
66	167 (9.3)	2640	
67	100 (5.6)		

*FG* fasting glucose, *Asp* Insulin aspart, *RHI* regular human insulin, *LIRA* liraglutide, *Lis* Insulin lispro, *QD* daily, *TID* three times a day, *CGM* continuous glucose monitoring

**Fig. 2:**
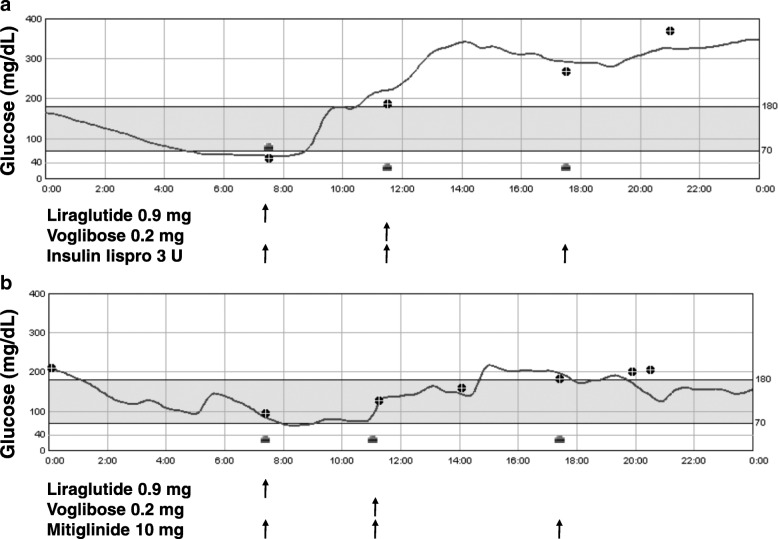
Glucose levels (mg/dL) determined with a continuous glucose monitoring system for 24 h, with treatment (**a**) insulin lispro 3 U before meals, liraglutide 0.9 mg before breakfast and voglibose 0.2 mg before lunch and (**b**) mitiglinide 10 mg before meals, liraglutide 0.9 mg before breakfast and voglibose 0.2 mg before lunch.  represents the time of each meal.  represents capillary glucose level for sensor signal calibration

**Fig. 3:**
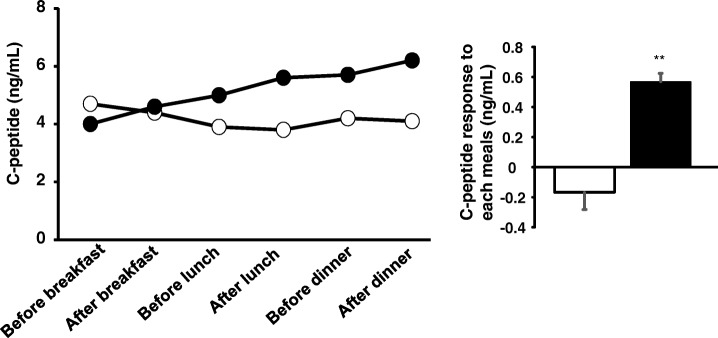
Diurnal C-peptide levels before and 2 h after each meal and C-peptide responses to each meal when given liraglutide 0.9 mg before breakfast, voglibose 0.2 mg before lunch and insulin lispro 3 U before each meal at the 54th day after hospitalization (white circle or bar) and liraglutide 0.9 mg before breakfast, voglibose 0.2 mg before lunch and mitiglinide 10 mg before each meal at the 59th day after hospitalization (black circle or bar) The data on C-peptide responses to each meal are presented as postprandial C-peptide increments ± SD (*n* = 3). **, *P* < 0.01. The results of comparisons of treatments were assessed employing the unpaired t-test

First, liraglutide, and then voglibose were added to insulin treatment, but continuous glucose monitoring (CGM) using iPro2® (Medtronic Japan, Tokyo, Japan) revealed that treatment with insulin lispro 3 U before each meal, liraglutide 0.9 mg before breakfast and voglibose 0.2 mg before lunch was not able to suppress either marked diurnal hyperglycemia or the early-morning hypoglycemia (Fig. 2a). Voglibose could not be used more than once a day due to exacerbation of diarrhea. The replacement of insulin lispro with mitiglinide before each meal effectively inhibited glycemic fluctuations. Typical CGM data are shown in Fig. 2b. The mean amplitude of glycemic excursion values for 72 h (analyzed on 51st-53rd and 61st-63rd days of hospitalization) was decreased from 216 mg/dL (12 mmol/L) to 157 mg/dL (8.7 mmol/L) by the replacement of insulin lispro with mitiglinide. To precisely evaluate endogenous insulin secretion, we used the Chemilumi C-peptide kit (Siemens Healthcare Diagnostics), which shows negligible cross-reactivity with proinsulin. Although serum C-peptide remained high due to renal failure, mitiglinide significantly raised serum C-peptide levels 2 h after, as compared with those before meals (Fig. 3), indicating increased postprandial insulin secretion. Furthermore, the replacement of insulin lispro with mitiglinide decreased insulin levels from 3500 μU/mL to 2640 μU/mL, despite the persistently high binding rate (84.2%), low affinity (K1 = 0.0236 × 10^8^ M^− 1^) and high capacity (R1 = 84.4 × 10^− 8^ M) of insulin antibodies (analyzed on the 61st day of hospitalization).

## Discussion and conclusions

In the present case, the IAS-like insulin antibodies with high binding capacity and low affinity may induce marked blood glucose fluctuations with frequent morning hypoglycemic attacks. In most IAS cases, administration of agents containing a sulfhydryl group, including methimazole and α-lipoic acid [10, 11], is considered to trigger autoantibody production. However, our patient had not received such medications. A report indicated that switching to analog insulin from human insulin triggered IAS-like insulin antibody production [4]. In our present patient as well, hypoglycemic episodes manifested after one year of treatment with insulin analogs. In addition, this patient has HLA-DRB1*0406 which is associated with the highest risk of susceptibility to IAS [9]. These findings suggest that insulin analog treatment triggered the production of IAS-like insulin antibodies, leading to blood glucose fluctuations. Since this patient had low insulin secretory capacity and insulin resistance, he might have been prone to hypoglycemic episodes in fasting compared with postprandial states.

He did not require insulin therapy and his endogenous insulin secretion was essentially maintained (fasting serum C-peptide: 1.41 ng/mL) 15 years after the onset but his insulin secretory capacity gradually decreased. In making the differential diagnosis, latent autoimmune diabetes in adults should be considered [12]. However, the islet-related “auto”-antibodies that we measured, such as those against GAD and IA-2, were all negative, though IAS-like insulin antibody was detectable. Based on these clinical findings, we diagnosed this patient as having type 2 diabetes.

For IAS patients, consuming small meals and α-glucosidase treatment to decrease insulin secretion are recommended for suppressing both hyperinsulinemia and hypoglycemia [2].

However, in this patient, larger amounts of postprandial insulin than he had secreted were needed to prevent hyperglycemia even after α-glucosidase treatment. Therefore, we had to determine which therapy, exogenous injection of insulin or augmentation of endogenous insulin secretion, was better for ameliorating glycemic fluctuations. We speculated that exogenously injected insulin, retained subcutaneously, might have more chances to be bound by insulin antibodies. In addition, endogenous insulin directly enters the portal vein and suppresses hepatic glucose production. Portal insulin reportedly exhibits several times higher than in the peripheral arterial circulation in the steady state [13, 14]. Exogenous insulin cannot recreate this physiological portal-systemic difference in the insulin distribution, seemingly resulting in inappropriately high insulin levels at the periphery. In fact, impaired suppression of hepatic glucose output is one of the most important mechanisms involved in postprandial hyperglycemia in patients with diabetes [15, 16]. Thus, excessive peripheral insulin captured by IAS-like insulin antibodies may induce not only hypoglycemia but also postprandial hyperglycemia. Furthermore, patients with chronic renal failure are reportedly more susceptible to hypoglycemia, because of impaired insulin clearance rates [17]. Therefore, treatment strategies aimed at minimizing exogenous subcutaneous insulin were selected.

First, we used liraglutide to delay gastric emptying, and then added voglibose to delay glucose absorption, both of which were administered with the aim of decreasing the postprandial insulin requirement. However, glycemic fluctuations persisted even after these treatments. C-peptide responses after meals were not detected, although the longer half life of peripheral C-peptide due to renal failure might have masked small responses. In contrast, addition of mitiglinide, which is a rapid and short-acting insulin secretagogue, apparently increased postprandial C-peptide and dramatically attenuated the glycemic fluctuations. Furthermore, fasting insulin levels were decreased after mitiglinide administrations as compared with those during insulin administrations. Thus, enhanced postprandial endogenous insulin secretion induced by mitiglinide achieved better glycemic control, due possibly to the efficient hepatic action of insulin and the alleviation of peripheral hyperinsulinemia.

Proper enhancement of postprandial endogenous insulin effectively suppresses postprandial hyperglycemia by decreasing both hepatic glucose release and dietary glucose outflow to the peripheral circulation, thereby constituting a potentially useful treatment for unstable diabetes induced by IAS-like insulin antibodies. When we encounter insulin-treated patients who exhibit glycemic instability and are positive for insulin antibodies, we should be careful to not simply rely on adjustment of insulin dosages. We should also consider enhancing endogenous insulin secretion to prevent hypoglycemic episodes.

## Abbreviations

CGM: Continuous glucose monitoring
HLA: Human leukocyte antigen
IAS: Insulin autoimmune syndrome

## References

[1] Hirata Y, Uchigata Y (1994). Insulin autoimmune syndrome in Japan. Diabetes Res Clin Pract.

[2] Uchigata Y, Hirata Y. Insulin autoimmune syndrome (IAS, Hirata disease). Ann Med Interne. 1999;150:245–53. 10445096

[3] Fineberg SE, Huang J, Brunelle R, Gulliya KS, Anderson JH (2003). Effect of long-term exposure to insulin lispro on the induction of antibody response in patients with type 1 or type 2 diabetes. Diabetes Care.

[4] Ishizuka T, Ogawa S, Mori T, Nako K, Nakamichi T, Oka Y, Ito S (2009). Characteristics of the antibodies of two patients who developed daytime hyperglycemia and morning hypoglycemia because of insulin antibodies. Diabetes Res Clin Pract.

[5] Iizuka K, Tomita R, Horikawa Y, Takeda J (2012). A case of glycemic instability and insulin allergy due to anti-insulin antibodies in a patient with type 2 diabetes. Diabetol Int.

[6] Tamura Y, Kimbara Y, Funatsuki S, Mabuchi S, Remi K, Yoshimoto A (2013). A case of insulin antibody-induced glucose instability in an elderly woman with type 2 diabetes on hemodialysis, successfully ameliorated with liraglutide. Diabetol Int.

[7] Lathela JT, Knip M, Paul R, Antonen J, Salmi J (1997). Severe antibody-mediated human insulin resistance: successful treatment with the insulin analog Lispro. Diabetes Care.

[8] Moriyama M, Hayashi N, Ohyabu C, Mukai M, Kawano S, Kumagai S (2006). Performance Evaluation and Cross-reactivity from insulin analogs with the ARCHITECT insulin assay. Clin Chem.

[9] Uchigata Y, Tokunaga K, Nepom G, Bannai M, Kuwata S, Dozio N (1995). Differential Immunogenetic determinants of polyclonal insulin autoimmune syndrome (Hirata's disease) and monoclonal insulin autoimmune syndrome. Diabetes.

[10] Uchigata Y, Hirata Y, Iwamoto Y (2009). Drug-induced insulin autoimmune syndrome. Diabetes Res Clin Pract.

[11] Yamada T, Imai J, Ishigaki Y, Hinokio Y, Oka Y, Katagiri H (2007). Possible relevance of HLA-DRB1*0403 haplotype in insulin autoimmune syndrome induced by alpha-lipoic acid, used as a dietary supplement. Diabetes Care.

[12] Buzzetti R, Zampetti S, Maddaloni E (2017). Adult-onset autoimmune diabetes: current knowledge and implications for management. Nat Rev Endocrinol.

[13] Song SH, Mcintyre SS, Shah H, Veldhuis JD, Hayes PC, Butler PC (2000). Direct measurement of pulsatile insulin secretion from the portal vein in human subjects. J Clin Endocrinol Metab.

[14] Horwits DL, Starr JI, Mako ME, Blackard WG, Rubenstein AH (1975). Proinsulin, insulin, and C-peptide concentrations in human portal and peripheral blood. J Clin Invest.

[15] DeFronzo RA (1992). Pathogenesis of type 2 (non-insulin dependent) diabetes mellitus: a balanced overview. Diabetologia.

[16] Rizza RA (2010). Pathogenesis of fasting and postprandial hyperglycemia in type 2 diabetes: implications for therapy. Diabetes.

[17] Moen MF, Zhan M, Hsu VD, Walker LD, Einhorn LM, Seliger SL (2009). Frequency of hypoglycemia and its significance in chronic kidney disease. Clin J Am Soc Nephrol.

